# Nerve-perivascular fat communication as a potential influence on the performance of blood vessels used as coronary artery bypass grafts

**DOI:** 10.1007/s12079-017-0393-7

**Published:** 2017-06-10

**Authors:** Andrzej Loesch, Michael R. Dashwood

**Affiliations:** 1 Centre for Rheumatology and Connective Tissue Diseases, Division of Medicine, University College London Medical School, Royal Free Campus, Rowland Hill Street, NW3 2PF, London, UK; 2 Division of Surgery and Interventional Science, Faculty of Medical Sciences, University College London Medical School, Royal Free Campus, Rowland Hill Street, NW3 2PF, London, UK

**Keywords:** Perivascular, Nerves, Adipose tissue, Bypass grafts, Vasoreactivity

## Abstract

Perivascular fat, the cushion of adipose tissue surrounding blood vessels, possesses dilator, anti-contractile and constrictor actions. The majority of these effects have been demonstrated in vitro and may depend on the vessel and/or the experimental method or species used. In general, the relaxant effect of perivascular adipose tissue is local and may be either endothelium-dependent or endothelium-independent. However, nerve stimulation studies show that, in general, perivascular adipose tissue (PVAT) has an anti-contractile vascular effect likely to involve an action of the autonomic vascular nerves. Apart from a direct effect of perivascular fat-derived factors on bypass conduits, an interaction with a number of neurotransmitters and other agents may play an important role in graft performance. Although the vascular effects of PVAT are now well-established there is a lack of information regarding the role and/or involvement of peripheral nerves including autonomic nerves. For example, are perivascular adipocytes innervated and does PVAT affect neuronal control of vessels used as grafts? To date there is a paucity of electrophysiological studies into nerve-perivascular fat control. This review provides an overview of the vascular actions of PVAT, focussing on its potential relevance on blood vessels used as bypass grafts. In particular, the anatomical relationship between the perivascular nerves and fat are considered and the role of the perivascular-nerve/fat axis in the performance of bypass grafts is also discussed.

## Coronary artery bypass grafts (CABG)

According to the most recent World Health Organisation report there are over 7 million deaths a year due to coronary heart disease (see WHO Cardiovascular diseases (CVDs) (2017) at http://www.who.int/mediacentre/factsheets/fs317/en/) worldwide with more than 800,000 coronary artery bypass graft (CABG) procedures performed annually. CABG surgery is an effective means of restoring blood supply to the myocardium in cases where flow through a coronary artery is reduced or blocked due to atherosclerosis. A number of different blood vessels have been used for revascularization including the internal thoracic artery (ITA), radial artery (RA), gastroepiploic artery (GEA) and the saphenous vein (SV). Currently the ITA, RA and SV are the most commonly used vessels and, of the three, the SV is the most frequently used. There are a number of reasons for this. The use of autologous grafts eliminates problems of tissue rejection and the need for tissue typing and matching. The SV also has a number of practical advantages: it is expendable, since lower limb venous drainage can rely solely on the deep venous system; its superficial position renders it easily accessible, facilitating its exposure at harvest (Favaloro 1969; Tsui and Dashwood 2002) and its long length allows its use for multiple grafts: “the average length of saphenous vein available (from each leg) when harvesting for coronary artery bypass procedures is 31 cm” (Human et al. 2009). Another factor contributing to the SV’s suitability as a graft includes its well-developed tunica media which is due to its lack of support by the tissue of surrounding structures and the need to withstand the hydrostatic pressures generated by the long column of blood when a person stands erect.

Human arteries are subjected to 60–140 mmHg pressures, pulsatile flow and a shear stress of ~3–6 dyne/cm^2^ whereas, in general, veins are subjected to lower pressures of ~5–8 mmHg, a non-pulsatile flow and a shear stress of ~0.2 dyne/cm (Lemson et al. 2000). However, unlike many commonly-used experimental animals where the venous system remains under fairly constant flow and pressure, human veins of the lower limbs are subjected to variable orthostatic pressures caused by alterations in posture and movement. For example supine and orthostatic venous pressures in healthy volunteers have been reported to be 7 ± 1 mmHg and 76 ± 2 mmHg respectively (Stick et al. 1993).

## Blood vessel structure

**Fig. 1:**
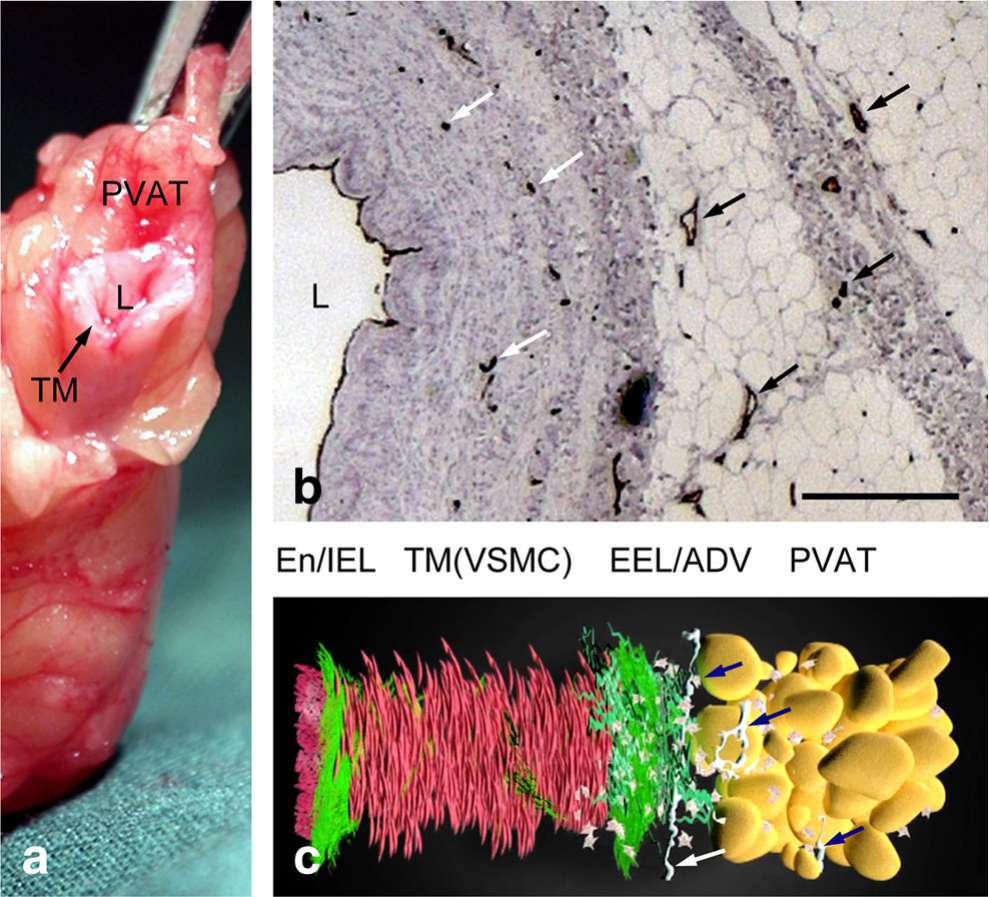
Structure of vessel wall of human saphenous vein. **a** A length of SV harvested in preparation for use as a graft in a patient undergoing CABG; L-lumen, TM-tunica media, PVAT-perivascular adipose tissue. **b** Light microscopy of a part of a transverse section of a human SV used for CABG immunolabelled for endothelial cells (CD31 antibody: *brown staining*) lining the lumen (L) and vasa vasorum (*arrows*) of the media, adventitia and PVAT (from MR Dashwood unpublished). Bar: 250 μm. **c** Animated reconstruction of a human SV used for CABG showing the layers of the wall and nerves (*arrows*); End/IEL-endothelium/internal elastic lamina, TM(VSMCs)- vascular smooth muscle cells of the tunica media; EEL/ADV-external elastic lamina/adventitia. (We gratefully acknowledge Dr. Craig Daly and Ms. Anna Mikelsone at www.cardiovascular.org for permission to use this image; see Mikelsone (2014))

Most large diameter blood vessels are characterised by three main layers (‘tunicas’); the intima, the media and the adventitia. The muscular arteries used as bypass grafts have a well-defined internal elastic lamina (IEL) at the inner region of the tunica intima and a diffuse external elastic lamina (EEL) bordering the media and adventitia. The intima consists of a thin basement membrane and its endothelial lining. Elastic fibres are scattered within the media, which is mainly composed of vascular smooth muscle cells (VSMCs) and collagen. A microvascular network, the vasa vasorum, is mainly confined to the outer adventitial layer although extending into the media. Also, mainly located in the adventitia, are the autonomic or perivascular nerves. The human SV, which is a subject of particular interest here, has a muscular wall consisting of several layers of VSMCs separated by layers of collagenous connective tissue. The tunica media and intima also contain a few elastic fibres but there is no distinct IEL or EEL as in arteries of similar size. The outermost layer, the adventitia, is broad consisting mainly of collagen fibres and fibroblasts that merge with the surrounding connective tissue and perivascular adipose tissue (PVAT) (Fig. 1). The vasa vasorum in the SV are more pronounced and penetrate deeper into the media than in arteries. In medium- and large-sized (non-distended) veins such as the SV, the intima is thrown into folds and may be thickened in areas due to the presence of smooth muscle cells and collagen within regions of neointimal hyperplasia (Ham 1974).

## Perivascular adipose tissue (PVAT)

Most blood vessels in the body, apart from the brain, are embedded in, or surrounded by, fat – the tunica adiposa (Chaldakov et al. 2012, 2014) that is often also implicated in inflammation and cardiometabolic diseases (Blirando 2016). PVAT, that surrounds most blood vessels, is no longer thought of as merely having a ‘supporting’ role but is now recognised as being a source of factors that potentially influence vascular tone and structure (Szasz and Webb 2012). PVAT is comprised of discrete adipocytes containing a network of capillaries and nerve fibres as well as mast cells, macrophages, adipocyte stem/progenitor cells, lymphocytes, fibroblasts and myofibroblasts (Thureson-Klein and Stijӓrne 1979; Hu et al. 2004; Ruan et al. 2010; Campbell et al. 2012; Simerman et al. 2014). PVAT also plays a role in VSMCs growth (Miao and Li 2012). Apart from these features, PVAT appears sensitive to endovascular injury such as percutaneous transluminal angioplasty (experimental models of injury of mouse femoral artery and rat carotid artery), and undergoes rapid phenotypic changes (Takaoka et al. 2010).

## Vascular effects of PVAT

**Fig. 2:**
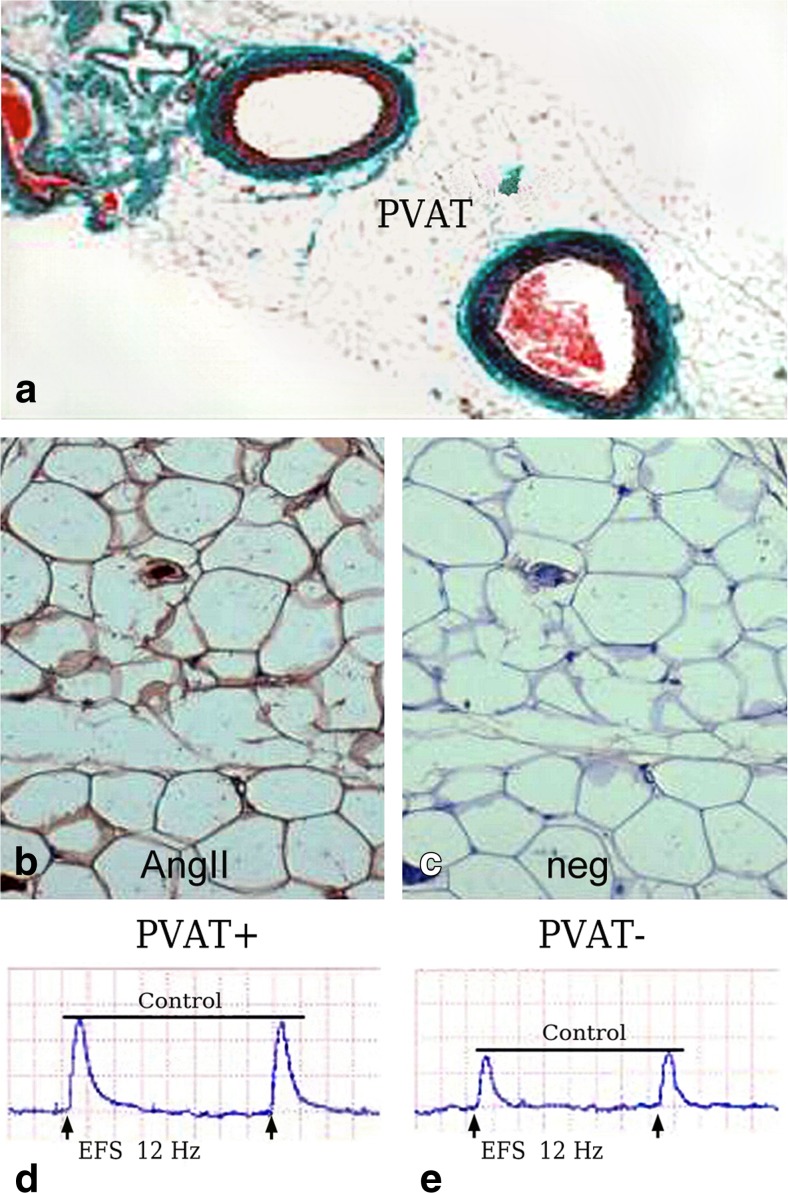
Location of angiotensin II (AngII) in PVAT and its effect on nerve stimulation. **a** Sections of rat mesenteric arteries surrounded by PVAT. **b** Presence of AngII by immunohistochemistry (*brown stain*); **c** negative control (neg) for AngII staining. **d-e** Representative constrictor responses to electrical field stimulation (EFS) in arteries with PVAT intact (PVAT+) and PVAT removed (PVAT-); there is attenuation of the constrictor effect when PVAT is removed. (**a-e** Modified from Lu et al., Eur J Pharmacol 2010, 634(1–3):107–112 [Elsevier] with permission, which we gratefully acknowledge)

PVAT is now accepted as a dual modulator of vascular function where attenuation of contractile responses to agonists by PVAT involves both an endothelium-dependent mechanism via the release of a transferable relaxation factor(s) (Löhn et al. 2002; Gao et al. 2005) and an endothelium-independent mechanism involving hydrogen peroxide (Gao et al. 2007). However, PVAT also potentiates vasoconstriction to perivascular neuronal excitation by electrical field stimulation (EFS) as shown in rat mesenteric arteries, a mechanism involving superoxide production and subsequent activation of the MAPK/ERK pathway (Gao et al. 2006). Using the same rat PVAT mesenteric artery model, this group also showed, using vessels treated with an ACE inhibitor (enalaprilat) or angiotensin II type 1 receptor antagonist (candesartan), that adipocyte-derived angiotensin II is critically involved in PVAT-mediated potentiation of EFS-evoked vascular contraction. In support of the functional studies, the presence of angiotensinogen and angiotensin I-converting enzyme (ACE) mRNA was confirmed by RT-PCR and immunohistochemical staining showed the presence of angiotensin II in mesenteric PVAT. It was concluded that these results showed that adipocyte-derived angiotensin II is critically involved in PVAT-mediated potentiation of EFS-evoked contraction in rat mesenteric arteries (Lu et al. 2010). Figure 2 illustrates the above-discussed phenomena in mesenteric artery model related to PVAT, angiotensin II and EFS. The effects of PVAT on sympathetic and sensory perivascular neurotransmission on rat mesenteric arterial beds with and without PVAT have also been reported (Bakar et al. 2013). Here EFS was performed at different frequencies in the absence/presence of pharmacological agents in an attempt to discriminate between neurogenic dilator and constrictor responses. The final conclusion of the authors was that the presence of PVAT modulated responses to activation of both sympathetic and sensory nerves (Bakar et al. 2013). More detailed studies by this group revealed the functional expression of sensory nerves within PVAT and that PVAT was essential for sensory neurogenic vasorelaxation via calcitonin gene-related peptide (CGRP) and crosstalk with adipocytes leading to leptin release (Abu Bakar et al. 2017).

An electrophysiological study using the isolated cat SV examined the response of primary afferent neurons to both mechanical and chemical stimulation. Here, these nerves responded to both chemical (hypertonic saline, bradykinin and capsaicin) and mechanical (intraluminal pressures ranging from 35 to 250 mmHg) stimuli. A small proportion of vein afferent nerves responded to ‘noxious (and/or chemical) stimuli’ suggesting that they may potentially encode nociceptive information from the vein, especially under pathological conditions (e.g. such as the increased inflammatory cytokine levels that occurs in failing bypass grafts) (Michaelis et al. 1994). Regarding the autonomic control of the SV, neural connections identified using the retrograde tracer horseradish peroxidase, located sympathetic postganglionic neurons innervating the femoral-saphenous vein that arose from lumbar regions of the spinal cord (Chen and Liu 1993).

Although many in vitro and in vivo studies have described various aspects of autonomic vasomotor control and other effects of PVAT on vessel structure and function, as far as we are aware few have focussed on perivascular nerve-adipose tissue communication. In the next section of this review anatomical data is presented showing potential sites connecting autonomic nerves and PVAT.

## Light microscopic observations

**Fig. 3:**
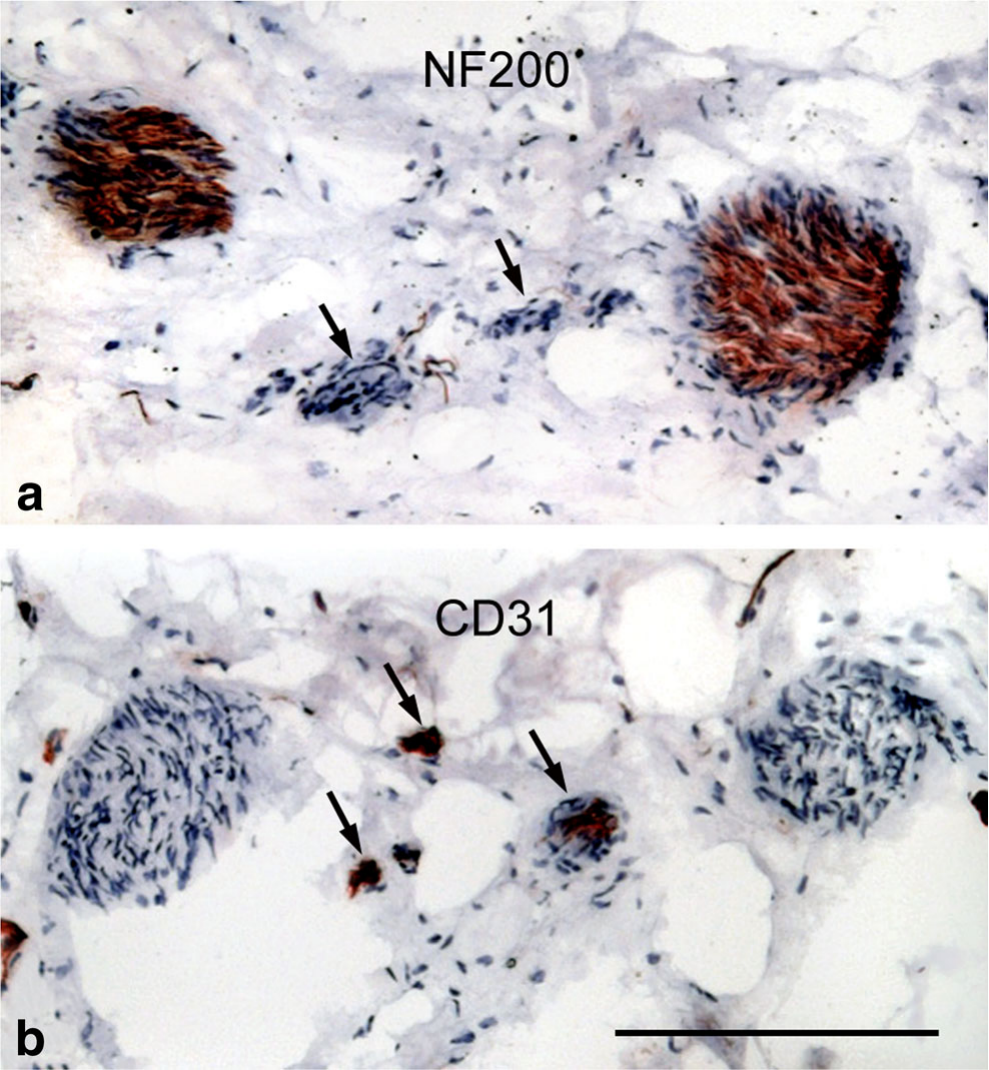
Close proximity of paravascular nerves and vasa vasorum at the border of the adventitia and PVAT of human saphenous vein. **a** Nerves identified by immunohistochemistry using an antibody to NF200 (*brown staining*). Arrows show the position of a vasa vasorum (identified in the **b**). **b** Endothelial cells of the vasa vasorum identified by immunohistochemistry using an antibody to CD31 (*arrows - brown staining*). The sections were counterstained with haematoxylin. Bar: 50 μm. (MR Dashwood unpublished)

There is limited information on nerve/nerve-fat localization or distribution in standard text books. This is mainly due to lack of nerve-specific stains in the past with the introduction of the Falck-Hillarp catecholamine-induced fluorescence technique (Carlsson et al. 1962) providing early data describing the distribution of nerves mainly confined to the adventitia/perivascular regions. The introduction of nerve-specific antibodies, such as anti-protein gene product (PGP) 9.5 and anti-neurofilament 200 (NF200), presented the ability to study, not only nerve distribution in nerves (nerve nervorum), but also to identify neurotransmitters and/or co-transmitters affecting vessel reactivity such as CGRP, substance P (SP), vasoactive intestinal polypeptide (VIP), endothelin (ET) and nitric oxide (NO). Regarding vascular/nerve studies on human blood vessels, neuropeptide Y (NPY) has been shown to co-exist with noradrenergic neurones in skeletal blood vessels of experimental animals and human (Pernow et al. 1987) and CGRP, SP, VIP and NPY have been identified in various regions of human epicardial coronary arteries (Gulbenkian et al. 1993). Of relevance to this review is the observation that perivascular nerves and vasa vasorum of SVs used as grafts in patients undergoing CABG displayed staining for nitric oxide synthase, suggesting that nitrergic nerves may play a role in vasomotor control of this vessel and that this may be affected when the SV is removed and used as a graft (Tsui et al. 2002). Within the outermost layers of both coronary arteries and vessels used as bypass conduits the perivascular nerves and vasa vasorum are in close proximity to one other (Fig. 3). Apart from the endothelium-derived peptide, ET-1, being a potent vasoconstrictor, there is some evidence that it may also have neurotransmitter or co-transmitter activity (Wiklund et al. 1988; Nakamaru et al. 1989; Wong-Dusting et al. 1989; Tabuchi et al. 1989, 1990; see also Dashwood and Loesch 2010). In an early study, following the isolation and identification of ET-1, the distribution of its binding sites (putative receptors) in human and porcine vessels were described that was particularly dense in the media with clusters of perivascular binding to nerve trunks with the authors suggesting that ET-1 may act as a neuromodulator of noradrenergic transmission (Power et al. 1989). In a later study on human and porcine coronary arteries and human SV, a most striking ET-1 binding was observed that was associated with the vasa vasorum and regions of neovascularization of atherosclerotic coronary arteries (Dashwood et al. 1993). In a porcine vein graft model ET-1 receptors were identified in the adventitia of SV grafts and were associated with regions of neovascularization of occluding grafts (Dashwood et al. 1998a, b) and, in a later study, to regions of neural reorganization in the adventitia (Dashwood et al. 2000). These results on the porcine vein graft model and on SV obtained from patients undergoing CABG are taken as evidence that ET-1 may play both vascular and neural roles in vein graft failure (Dashwood 2009).

## Human SV vasa vasorum and autonomic innervation: General

As has already been mentioned, vasa vasorum of human SV is a complex heterogeneous system originating from feeding blood vessels – both artery and vein - giving rise to vessels of various diameter including arterioles, venules and capillaries (Lametschwandtner et al. 2004; Kachlík et al. 2003; Kachlík et al. 2007). It has also been shown that the human SV vasa system is extensive and significantly more prolific compared to that in other vessels used for CABG, for example the ITA and RA (Dreifaldt et al. 2011, 2012). The autonomic nervous system is generally involved in a plethora of physiological and pathophysiological processes, including those affecting the control of the tone of VSMCs and therefore also implicated in local blood flow control (Ralevic and Burnstock 1993, 1995). Like other large vessels in the body, the human SV is innervated by the autonomic sympathetic, parasympathetic and sensory nerves (Amenta et al. 1983; Herbst et al. 1992; Fabi et al. 1993; Racchi et al. 1999; Loesch and Dashwood 2009). Of the nerves in human SV it is the sympathetic nerves that have attracted the most attention (Fabi et al. 1993; Racchi et al. 1999).

## Human SV: Sympathetic nerves

We focus here on sympathetic nerves as these interact with both VSMCs and PVAT (Bartness and Bamshad 1998; Bulloch and Daly 2014) and have been observed by various histological means, for example in human SV used for CABG (Dashwood and Loesch 2011). It is well recognised that the sympathetic nerves release adenosine 5′-triphosphate (ATP) and noradrenaline (NA) as co-transmitters at various targets, together with NPY, which usually acts as a prejunctional modulator (Burnstock 2007; Ralevic and Dunn 2015). This complex sympathetic signaling, which involves specific receptors, is also true for the sympathetic perivascular innervation of human SV samples from patients undergoing cardiac revascularization (Fabi et al. 1993; Rump and von Kügelgen 1994; Racchi et al. 1999); in such SV samples, activation of calcium channels modulates the contraction of VSMCs (Fabi et al. 1993). The same might be true for the VSMCs of SV vasa vasorum since SV vasa vessels are richly innervated by sympathetic nerves (Loesch and Dashwood 2009a). Therefore the sympathetic perivascular transmission to SV VSMCs, including those VSMCs in the vein vasa vasorum, might be crucial both for the vasoconstrictor control of SV tone and for blood flow through the vasa vasorum. Interestingly, according to Crotty (1992), a localized venodilator feedback phenomenon may also occur in certain situations, where the venoconstrictor effect of NA released from sympathetic perivascular nerves is reduced by NA circulating in the vasa vessels, as observed both in *in situ* and in vitro preparations of canine SV (Crotty 1992, 2007). Here, it is noteworthy that the responsiveness of human SV and its receptors to vasoactive agents/transmitters may change in pathophysiological conditions (Brunner et al. 2001; Ziganshin et al. 2003, 2004). In the context of sympathetic signaling, P2 (mostly P2X_1_) receptor-mediated contractions evoked by ATP are significantly lowered in varicose SVs compared to those from patients with obliterating atherosclerosis; it has been suggested that P2-receptors may therefore be involved in the pathogenesis of varicose vein disease (Ziganshin et al. 2003). Indeed, there are changes in the expression of ATP receptors in patients’ SV during varicose disease (Metcalfe et al. 2007). Our own confocal microscope observations of human SV harvested for CABG suggest that P2X_1_ receptors are widely distributed in circular and longitudinal layers of VSMCs of the media (Loesch and Dashwood 2009a).

**Fig. 4:**
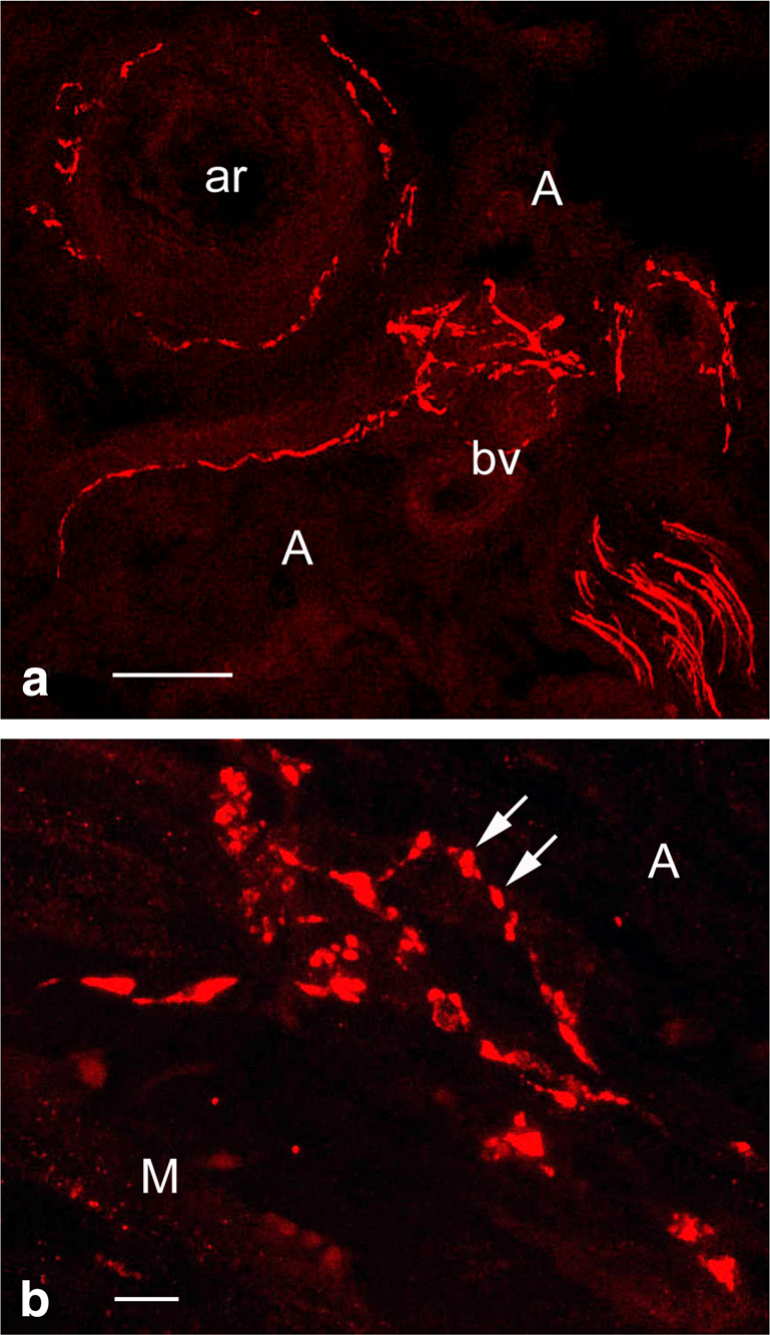
Confocal microscopy of 30 μm frozen cross-sections of non-stripped and non-distended (control) human SV graft preparations for CABG (~ 30 min to harvesting) with preserved paravascular connective tissue including white adipose tissue displaying immunolabelling for tyrosine hydroxylase (TH) in sympathetic nerve fibres (*red*). **a** Note sympathetic nerve fibres in the outer adventitia (A) and in the vicinity of vasa blood vessels (bv) including an arteriole (ar). Bar: 50 μm. **b** At higher magnification note varicosities (arrows) of sympathetic nerve fibres at the adventitia (A) – media (M) border. Bar: 10 μm. [Note that a rabbit polyclonal anti-TH antibody (TZ 1010, Affinity, Exeter, UK) was used at 1:300 dilution. Goat anti-rabbit Alexa 568 (Molecular Probes, Oregon, USA) was used at 1:600 as a second layer. Confocal laser microscope: BioRad Radiance 2000]. (We gratefully acknowledge that this figure was modified from Loesch and Dashwood, Phlebology Digest [Excerpta Medica Elsevier BV, Amsterdam] 2009b, 22(2):22–24)

**Fig. 5:**
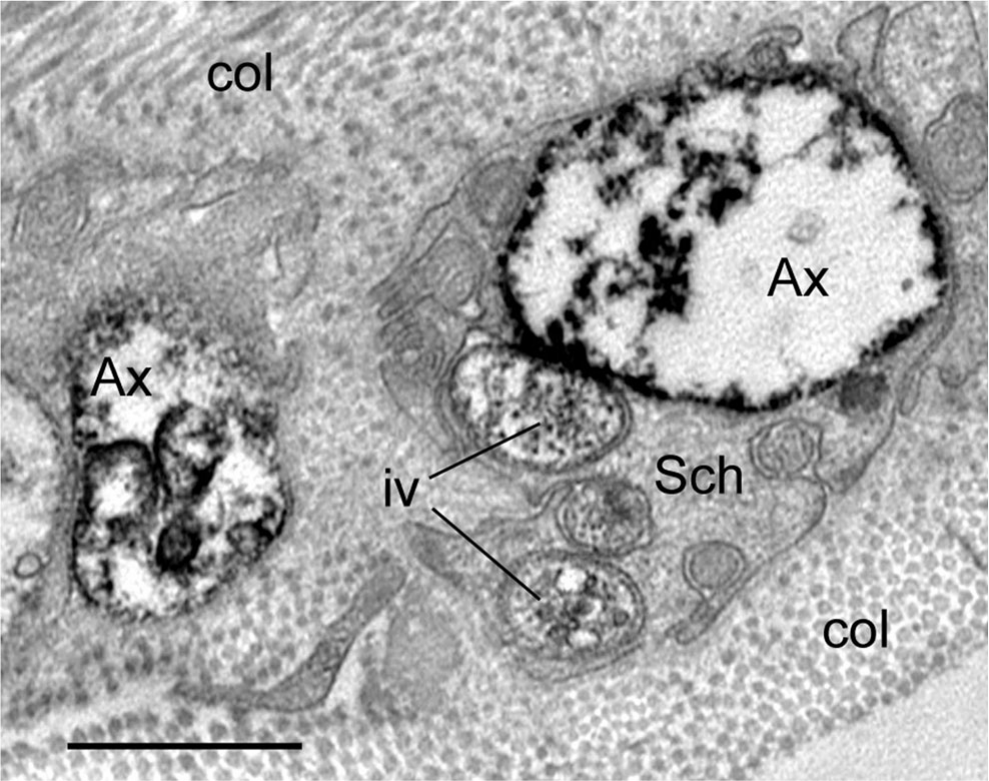
Transmission electron microscopy of perivascular nerve fibres of human SV ~ 30 min to harvesting (vein partially stripped off pedicle) showing immunolabelling for tyrosine hydroxylase (*black stain*) in sympathetic nerves. Note that the axon varicosities (Ax) of sympathetic nerves are damaged displaying partially “empty” axoplasm; immune-positive intervaricosities (iv) and immune-negative Schwann cell (Sch) are rather well preserved; col.-collagen. Similarly damaged axon varicosities can also be observed in non-stripped and non-dilated SV during harvesting; the proportion of axons affected in each method of harvesting is unknown. Bar: 1 μm. [Note that a TH rabbit polyclonal antibody (TZ1010, Exeter, UK) was used in the pre-embedding ExtrAvidin immunohistochemical method on paraformaldehyde-glutaraldehyde-fixed 50 μm cryostat sections, which then were embedded in Araldite; ultrathin 80 nm sections were examined with a Philips CM120 transmission electron microscope]. (We gratefully acknowledge that this figure was modified from Loesch and Dashwood, Phlebology Digest [Excerpta Medica Elsevier BV, Amsterdam] 2009b, 22(2):22–24)

As an example, here we demonstrate sympathetic innervation of the vasa vessels in the outer adventitia of human SV harvested for CABG (Fig. 4a); this is an example of perivascular nerves at the confocal microscope level following immunolabelling for tyrosine hydroxylase (TH), the rate limiting enzyme in NA synthesis (Levitt et al. 1965; Pickel et al. 1975). Observation of sympathetic perivascular innervation of the SV at higher magnification of such images distinguishes characteristic nerve/axon varicosities (Fig. 4b), generally known to be sites of neurotransmission to VSMCs (Burnstock 1988a, b). Sympathetic nerves of the human SV can also be identified at the electron microscope level following immunolabelling for TH, e.g. by the display of electron-dense (“black”) immunoprecipitate (Fig. 5). Some of the TH-positive axons in a SV used for CABG are ultrastructurally well-preserved, but some display structural alterations. This might be the effect of vein denervation and other manipulations during harvesting for CABG. Indeed standard electron microscope observations revealed axon profiles including axon varicosities displaying a variety of morphological/ultrastructural characteristics, in particular in conventional SV graft preparations (Ahmed et al. 2004). On the other hand this nerve polymorphic phenomenon might not be entirely surprising as autonomic nerves, including perivascular nerves, are generally very plastic and their structure may vary in both physiological and pathophysiological conditions (Cowen and Burnstock 1986; Burnstock 2009). In fact, SV varicosities display small and large agranular and granular vesicles or pleomorphic vesicles, in particular in conventional SV preparations (Ahmed et al. 2004). Following the original SV denervation during harvesting for CABG, the fate of sympathetic nerves as well as parasympathetic and sensory nerves in the SV wall (or wall of RA or ITA for CABG) is unknown once the vein is implanted into the coronary vasculature. However, there is data indicating that the process of re-innervation of the coronary graft/s may take place after grafting as has been revealed in studies using a porcine vein graft model (Dashwood et al. 1998a, b). It has been shown, for example, that the re-innervation of the coronary epicardial artery arises after angioplasty injury, as also occurs after SV graft implantation into the coronary vasculature: a striking increase in paravascular innervation/reinnervation as identified by the nerve marker NF200. Furthermore, nerves in SV grafts were primarily located within the outer part of the neoadventitia (Dashwood et al. 1998a, b). Based on the results using NF200 immunolabelling combined with in vitro receptor autoradiographic localization of [^125^I]ET-1 receptor binding sites in epicardial artery following angioplasty, Dashwood et al. (1998a) suggest that ET-1 release from adventitial monocytes or fibroblasts might be increased and potentially promote vascular re-innervation. However, it should be pointed out that in human SV, binding sites for [^125^I]-ET-1 may dominate at regions of high VSMCs (the media) as well as at regions of high density vasa vasorum, and, in the case of diseased vessels, at regions of neovascularization (Dashwood et al. 1993). Furthermore, neovascularization and re-innervation of vasa vasorum can also occur on the side contralateral to the vascular injury as revealed by Milner et al. (1997) following balloon-catheter-induced injury to the rat carotid artery, where a transient increase in the density of sensory PGP-9.5-, SP- and CGRP-containing nerves innervating vasa vasorum in the contralateral, uninjured side was observed. This phenomenon is likely to reflect neurocompensatory responses to vascular injury (Milner et al. 1997).

## Ultrastructural observations: Fat-nerves-vasculature

**Fig. 6:**
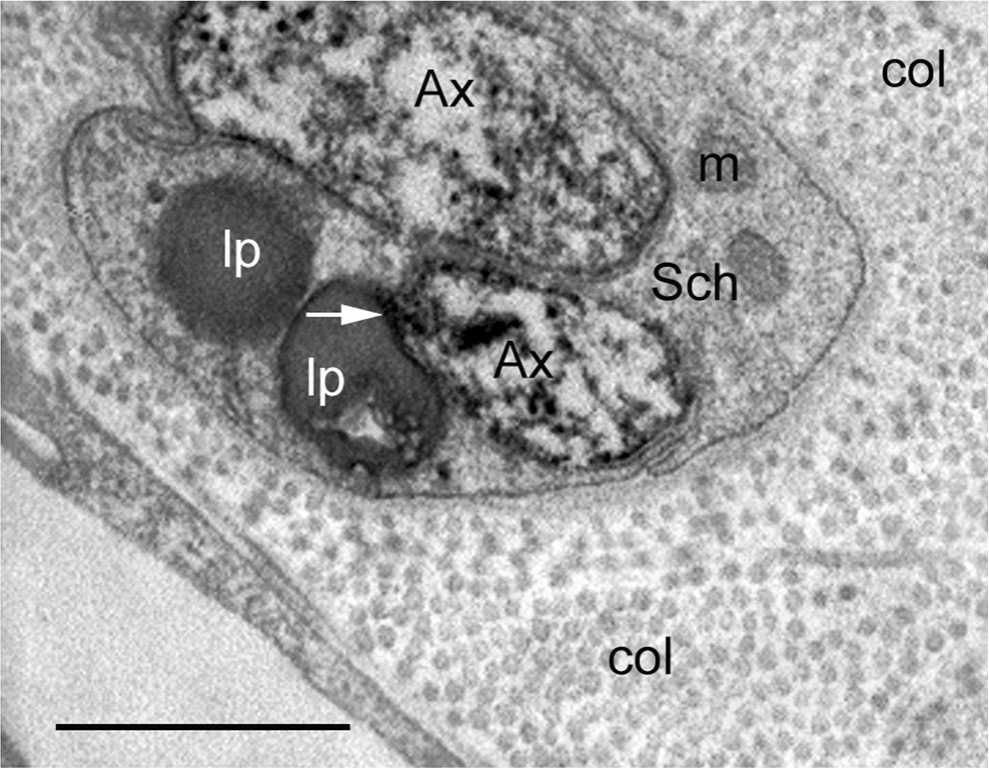
Transmission electron-immunocytochemistry of sympathetic perivascular nerve fibres of human SV (~ 30 min to harvesting) showing immunolabelling for TH (*black stain*) in two axon profiles (Ax) enclosed by a Schwann cell (Sch); Schwann cell displays two lipid droplets (lp). Note a close apposition (*arrow*) of a TH-positive axon profile and a lipid droplet; m-mitochondria, col.-collagen. Bar: 1 μm. [Note that the immunocytochemical method of TH detection was the same as described in legend to Fig. 2]. (A Loesch unpublished)

It has already been pointed out that, in addition to the effects on VSMC tone and hence the control of local blood flow (Ralevic and Burnstock 1993, 1995), the autonomic nervous system, including its sympathetic component, is in fact involved in a variety of physiological and pathophysiological processes (see Burnstock 2004, 2008; Ralevic and Dunn 2015; Burnstock and Loesch 2017). This also concerns the mutual relationship of the autonomic nervous system, and in particular, its sympathetic component with the PVAT (Ayala-Lopez et al. 2014; Bulloch and Daly 2014; Villacorta and Chang 2015; Ayala-Lopez and Watts 2016; Török et al. 2016; Ayala-Lopez et al. 2017). It appears that PVAT ‘recognises’ NA, NPY and ATP due to the expression of the relevant surface receptors on adipocytes (Bulloch and Daly 2014). Hence, NA from sympathetic nerves modulates lipolysis via α2- and β3 receptors, while it modulates lipogenesis via α1 and β3 receptors; NPY inhibits lipolysis via Y2 receptors; and ATP inhibits lipolysis via P2y receptors. It is therefore clear that sympathetic nerves impact on the contents of free fatty acids in PVAT (Bulloch and Daly 2014). Although there is no ultrastructural demonstration of a direct sympathetic interaction with PVAT in this review, we are able to demonstrate the possibility of interaction between the fat/lipids in Schwann cells and the associated sympathetic nerve/axons in human SV used for CABG (Fig. 6). The presence of lipids in Schwann cells is usually linked with the metabolic disturbances/pathology and/or neuropathy during degeneration and regeneration or myelin degradation as observed in myelinating Schwann cells (e.g. Goodrum et al. 1990, 1994). Lipids or their components can also be internalised by endocytosis by non-myelinating Schwann cells (Lee et al. 2013) where they are involved in lipid metabolism and where apolipoprotein E plays a central role (Boyles et al. 1985; Huang and Mahley 2014). The fact that the sympathetic nerve fibres can appear adjacent to lipid droplets in Schwann cells the question arises as to whether this phenomenon has a physiological effect on the nerve or vice versa? A recent study by Zeng et al. (2015) clearly shows that the lipolytic effect of leptin in mice subcutaneous inguinal fat and vasculature is mediated by sympathetic nerve fibres that innervate the adipose tissue. Hence, using intra-vital two-photon microscopy, these authors found that the sympathetic nerves project to white adipose tissue and form neuro-adipose junctions (Zeng et al. 2015).

Here, our own results, in particular from the human RA (see below), may indicate the existence of such a neuron/axon-adipose cell neuroeffector mechanism. Whether similar mechanisms can be related to the sympathetic nerve/axon contacting lipids in Schwann cells remains unknown at this stage. However, it has been shown that leptin can increase sympathetic efferent signalling to white adipose tissue to increase lipolysis (Scarpace and Matheny 1998; Rezai-Zadeh and Münzberg 2013). The above-mentioned data agrees with the observations of the association of TH/NPY-positive sympathetic nerves with adipose tissue (Giordano et al. 1996, 2005). In fact the nerve-adipose tissue association seems complex and heterogeneous. For example, projections of sensory nerves positive for CGRP and SP, as well as nerves (parasympathetic) positive for VIP have been observed in rat interscapular and periovarian adipose tissues (Himms-Hagen et al. 1990; Giordano et al. 1996). Interestingly, during prolonged fasting or environmental exposure to cold, an increase in sympathetic input to rat retroperitoneal and epididymal adipose tissue, its blood vessels and capillaries has been observed (Giordano et al. 1996, 2005).

**Fig. 7:**
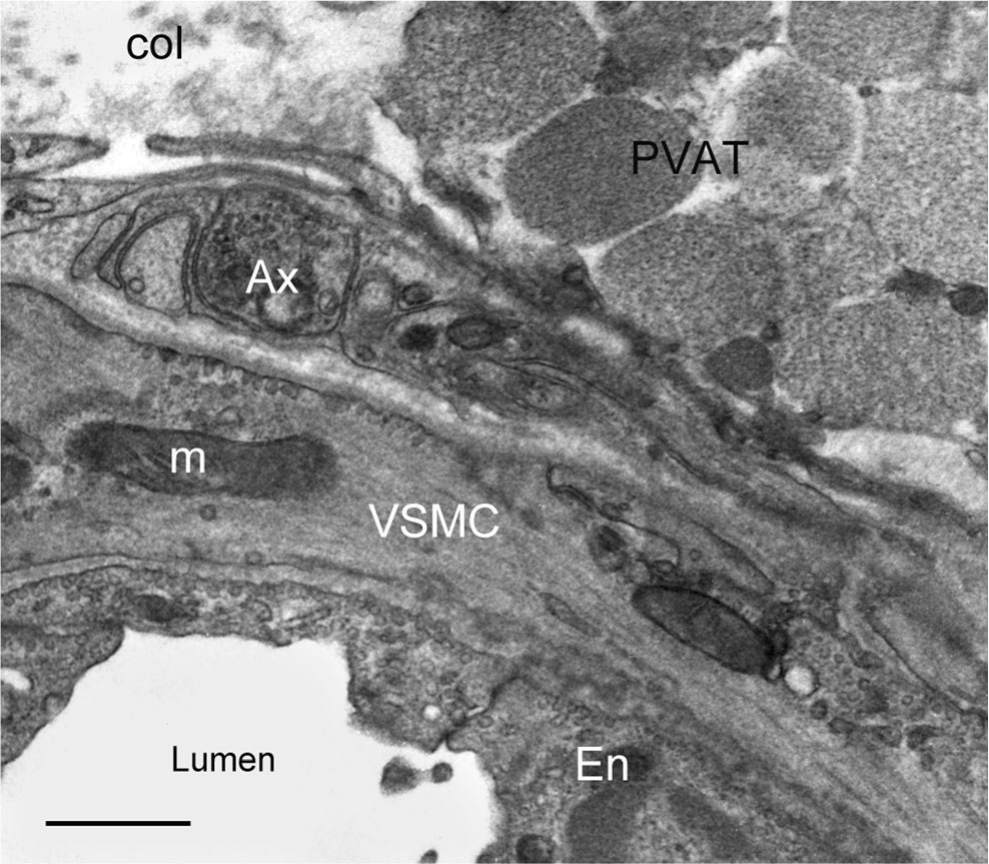
Standard transmission electron microscopy of a small artery (a branch of the Wistar rat right carotid artery); note association of adipose tissue (PVAT) with the artery wall, where perivascular nerves including axon varicosity (Ax) are also present; VSMC-vascular smooth muscle, En-endothelium, m-mitochondria, col.-collagen. Bar: 1 μm. [Note that the specimen was paraformaldehyde-glutaraldehyde fixed; ultrathin sections were examined with a JEM 1010 transmission electron microscope]. (A Loesch unpublished)

**Fig. 8:**
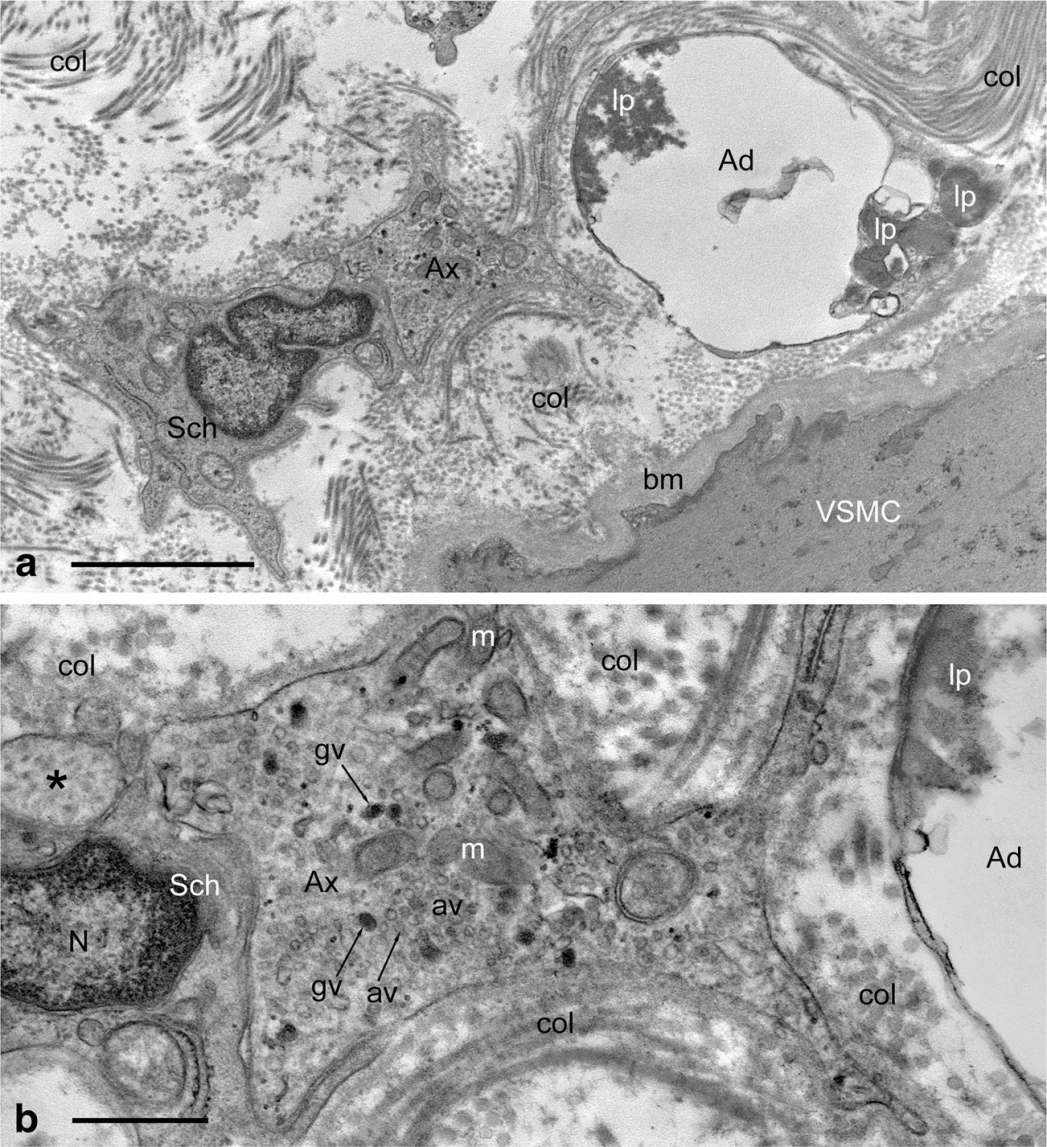
Standard transmission electron microscopy of a fragment of perivascular region of human RA harvested for CABG. **a** Note mutual arrangement of adipose tissue/cell (Ad), perivascular nerve including its varicosity (Ax) and Schwann cell (Sch), and also the vascular smooth muscle (VSMC) of the artery wall; note about 4 μm distance between varicosity and vascular smooth muscle. Lipid droplets/material (lp) of moderate electron-density is seen within adipose cell; col.-collagen, bm-basement membrane. Bar: 2 μm. **b** Higher magnification of the axon varicosity (Ax) from (**a**) displays the presence of granular (gv) and agranular (av) vesicles and also numerous mitochondria (m). Also note that the distance between the varicosity and the adipose cell (Ad) is about 0.5 μm thus it is shorter than that noted between the varicosity and VSMC, and hence it is well within the distance limit allowed for establishing neuroeffector junction (see Burnstock 2004); Sch-Schwann cell, N-nucleus, asterisk-axon intervaricosity; col.-collagen of perivascular connective tissue. Bar 0.5 μm. [Note that the specimens were paraformaldehyde-glutaraldehyde fixed, and the ultrathin sections examined with a Philips CM120 transmission electron microscope]. (A Loesch unpublished)

The importance of PVAT having an effect on the vasculature, in particular on the coronary vessels of the heart, has recently been highlighted (Fernández-Alfonso et al. 2017). Here we show two ultrastructural examples of a possible interactions (via neuroeffector junctions) between PVAT and autonomic nerves both in animal (Fig. 7) and human (Fig. 8a, b) vasculature. It is therefore possible that perivascular nerve/axon varicosities in human RA might affect neighbouring PVAT and vascular wall/VSMCs simultaneously; such varicosities display a variety of intracellular structures including granular and agranular vesicles suggesting that these can release transmitters ‘*en passage*’ to evoke effects on PVAT and VSMCs (Fig. 8b).

## Conclusions

Data presented in this review point to a potentially complex anatomical, structural and physiological interrelationship between perivascular nerves and PVAT as well as the vasa vasorum in blood vessels used as coronary artery bypass grafts. This seems also true of some other human and animal blood vessels. A possible interaction between TH-positive sympathetic nerves and lipids in Schwann cells is also revealed in the human SV. Furthermore, ultrastructural features of a possible direct interaction between PVAT, autonomic nerves and the vasculature have been observed in human RA as well as in a small animal vessel – e.g. a branch of the rat carotid artery. In general the data accumulated to date suggests that there is a reciprocal interaction between PVAT and nerves and this may influence both vascular physiology and/or pathophysiology. Regarding the SV used in CABG, present data indicates that PVAT plays a protective role and that its removal has a detrimental effect on graft patency. The same may be true for arterial grafts that are generally harvested with PVAT intact. (Attempts to “replace” PVAT with an external stent are controversial as the stent may increase mechanical trauma to the graft and reduce its patency rate). Future functional/electrophysiological studies are required to determine the influence of the nerve-PVAT axis on graft performance. While a small number of in vitro studies on animal vessels have provided useful information, similar studies using human vessels are needed in an attempt to determine the functional relationship of the perivascular nerves and adipose tissue and the role this axis may play in the performance of bypass grafts.

## Abbreviations

ACE: angiotensin-converting enzyme
ADRF: adipocyte-derived relaxing factor
ATP: adenosine 5′-triphosphate
CABG: coronary artery bypass graft
CGRP: calcitonin gene-related peptide
EEL: external elastic lamina
EFS: electrical field stimulation
ET: endothelin
GEA: gastroepiploic artery
IEL: internal elastic lamina
ITA: internal thoracic artery
MAPK/ERK: mitogen-activated protein kinases/extracellular signal-regulated kinases
NA: noradrenalin
NF200: neurofilament 200
NO: nitric oxide
NPY: neuropeptide Y
PGP: protein gene product
PVAT: perivascular adipocyte tissue
PVF: perivascular fat
RA: radial artery
SP: substance P
SV: saphenous vein
TH: tyrosine hydroxylase
VIP: vasoactive intestinal polypeptide
VSMC: vascular smooth muscle cells
